# Suspected Interaction of Cranberry Juice Extracts and Tacrolimus Serum Levels: A Case Report

**DOI:** 10.7759/cureus.610

**Published:** 2016-05-16

**Authors:** Atman A Dave, Jones Samuel

**Affiliations:** 1 Medical Education, Saint Luke’s Hospital of Kansas City; 2 Nephrology, Renal Associates

**Keywords:** cranberry juice, drug interactions, cytochrome p450, tacrolimus

## Abstract

Cytochrome P450 inhibition through fruit supplement interactions often results in increased serum levels of calcineurin inhibitors, including tacrolimus. Cranberry extract is a supplement often used for the prevention of recurrent urinary tract infections (UTIs), which are common in renal allograft recipients. To our knowledge, a decrease in serum levels of tacrolimus as a result of cranberry extract interaction is unreported. A 40-year-old renal transplant patient taking cranberry extract capsules for her recurrent cystitis presented asymptomatically with low serum levels of tacrolimus. Dose increase had little effect on the level, and cessation of the cranberry extract returned levels to desired range. Cranberry extracts are an adjunctive therapy used in the management of recurrent UTIs. Tacrolimus, an immunosuppressive agent, is metabolized intestinally by isoenzymes of the P450 cytochrome. Cranberry extracts may alter this metabolism and lead to sub-therapeutic serum levels of tacrolimus. This interaction is heretofore unreported. Cranberry extracts should be carefully monitored in allograft recipients due to interactions with serum tacrolimus levels.

## Introduction

Recurrent urinary tract infections are common in renal allograft recipients, and epidemiological studies show a higher incidence of occurrence in recipients compared to the general population [1]. While there are several factors affecting risk, immunosuppression level plays a large role in the development of UTIs in this subset of patients [2]. Cranberry extracts and juices have shown to have modest effects in the prevention of recurrent urinary tract infections in women and children [3]. The speculative mechanisms for the protective effects of cranberry are attributed to the fruit’s proanthocyanidin content and its inhibition of uropathogenic adhesion to uroepithelium. Use of cranberry juices and extracts is widely considered a complementary medicine, and supplementation is often used in conjunction with antimicrobials or other therapies. However, drug interactions have been shown to arise in a number of fruit and herb-derived dietary supplements [4-5], and serum tacrolimus levels have been reported to be elevated in transplant patients following intake of grapefruit [6] and pomelo [7], The mechanism of this interaction has been characterized as an inhibition of the intestinal cytochrome P450 involved in preliminary metabolism of tacrolimus [8]. Here, we report a case of a patient with significantly depressed serum tacrolimus levels as a result of cranberry extract supplementation. Informed consent was obtained from the patient for this study.

## Case presentation

**Figure 1:**
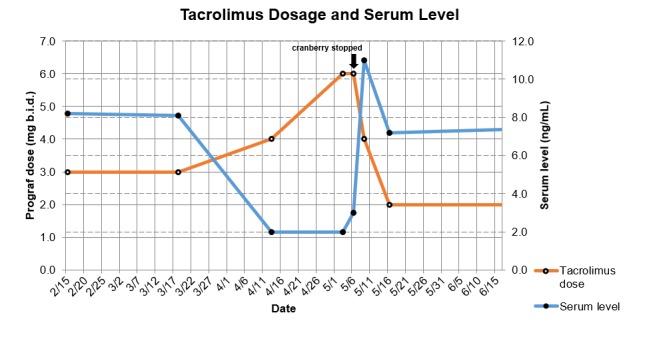
Tacrolimus Dosage and Serum Level

A 40-year-old woman presented with low serum levels of tacrolimus four years after undergoing live, related-donor renal transplantation for renal failure secondary to progressive systemic sclerosis. Her donor was a three human leukocyte antigen (HLA) match and was cytomegalovirus (CMV) positive. She was maintained on an immunosuppressive therapy of tacrolimus, mycophenolate mofetil, and prednisone, and her postoperative clinical course was uneventful with no rejection episodes. The patient experienced recurrent, posttransplant cystitis for which she was hospitalized on two occasions for intravenous antibiotic treatment with gram-negative and gram-positive coverage. She was subsequently referred to a urologist, whose workup did not reveal any anatomical cause. She was placed on prophylactic antibiotics (cephalexin, 500 mg q.d.) and cranberry juice extracts (1000 mg b.i.d.). Her serum tacrolimus level centered at 8.1 ng/mL prior to these treatments, and was in the acceptable range as depicted in Figure 1.

During a routine clinical visit, she was asymptomatic with normal allograft function, but had a markedly low trough level of tacrolimus (< 2.0 ng/mL) in a standard tacrolimus immunoassay. This was dismissed as possible technical error after the patient denied noncompliance with her current dose of tacrolimus (3 mg b.i.d.), which was subsequently increased to 4 mg b.i.d. The immunoassay was repeated and the level was again critically low (< 2.0 ng/mL), prompting urgent evaluation of the patient, which was unremarkable. At this point, the patient’s tacrolimus dose was doubled to 6 mg b.i.d. and the immunoassay repeated once more. The serum tacrolimus level increased to 3.0 ng/mL, but was still under the threshold of accepted immunosuppressive standards of 4.0 to 6.0 ng/mL.

After this finding, drug interactions were suspected as the patient denied any recent changes in diet or other habits. Interactions of the patient’s other medications listed in Table 1 were investigated, and only the cranberry juice extracts were a recent addition, with no described interactions. However, given the potential of fruit and herbal concentrates to alter the metabolism of tacrolimus, the cranberry juice extracts were withheld, and in conjunction her tacrolimus dosage was maintained at a level of 6 mg b.i.d. Her antibiotic prophylaxis for her recurrent cystitis was maintained given her history of infection. Her chemistries and evaluations were repeated, and her tacrolimus level was markedly higher at 11.0 ng/mL. Her dose was reduced to her previous 4 mg b.i.d., and then to 2 mg b.i.d, accompanied by return of stable serum tacrolimus levels (7.2 ng/mL). Throughout this episode, her allograft function remained normal with acceptable blood pressures and no proteinuria.

**Table 1:** Medications of the Patient

Medication	Dosage
Mycophenolic Acid	500 mg b.i.d.
Pantoprazole	40 mg b.i.d.
Tacrolimus	Varied
Prednisone	5 mg q.d.
Captopril	100 mg t.i.d.
Clonidine	0.1 mg q.d.
Venlafaxine	75 mg q.d.
Ondansetron	4 mg as needed
Acetaminophen	650 mg suppository q.i.d.
Cranberry Juice Extract	1000 mg b.i.d.
Tamsulosin	0.4 mg q.d.
Cephalexin	500 mg q.d.

## Discussion

Posttransplant cystitis is common in renal allograft recipients [3]. The evaluation of these patients involves a full urological workup to ensure proper bladder emptying and absence of strictures, stones, or other anatomical problems such as reflux, all of which may contribute to the recurrence of UTIs. In most cases, no definite cause is identified, and management involves education regarding proper personal hygiene, avoidance of constipation, probiotics, and prophylactic antibiotics [9].

Cranberry juices and extracts have been used as an adjunctive therapy in the management of patients with recurrent UTIs. In non-transplant patients, these extracts may have no consequence. Our case report suggests the supplements must be used with caution in transplant patients on tacrolimus, an immunosuppressive calcineurin inhibitor metabolized intestinally by isoenzymes CYP3A4 and CYP3A5, members of the P450 cytochrome.

Our patient had stable allograft function, had no evidence of gastrointestinal disturbances or malabsorption, and was on no other prescribed medications other than those listed in Table 1. The recent additions to this list were cephalexin (500 mg q.d.) and the cranberry extracts (1000 mg b.i.d). Cephalexin was not reported to have any drug interaction with tacrolimus, leading us to suspect the juice extract as the potential cause for the low serum concentrations of tacrolimus. This was further supported by the return of desired serum levels following discontinuation of the supplement.

Extensive review of the literature did not provide us with any direct reference to cranberry juice or cranberry extracts interacting with tacrolimus clinically, in vivo, or in vitro. However, cranberry has been shown to inhibit the intestinal CYP3A enzymes [10], which would lead to an increase in serum tacrolimus levels. All reported interactions with cranberry and CYP3A indicate increase of serum levels by this inhibition, the inverse of what is reported here. We suspect the cranberry extracts caused an induction of cytochrome P450 that would result in a low serum tacrolimus level; however we are unable to determine if the strength or composition of the cranberry juice extract had any role in the interaction. The contents of cranberry extracts are unregulated and are likely entirely dependent on the manufacturing process. The exact causative agents and exact mechanisms are therefore speculative.

The Drug Interaction Probability Scale (DIPS) was used to assess the likelihood of a drug interaction between tacrolimus and the cranberry extracts. This case report scored a DIPS value of four placing the episode in the ‘possible’ range of drug interaction.

## Conclusions

UTIs are common in renal transplant recipients and given the popularity of cranberry supplementation as a seemingly benign prophylactic, further elucidation of this drug interaction is important to avoid potential serious complications such as rejection episodes due to lowered tacrolimus levels.

Patients in this subset should be monitored for interactions arising from cranberry extracts due to their suspected interference with cytochrome P450 enzymes. This information would benefit both general nephrologists and primary care physicians who are increasingly involved in the continued care of stable transplant patients.
